# Curcumin attenuates angiogenesis in liver fibrosis and inhibits angiogenic properties of hepatic stellate cells

**DOI:** 10.1111/jcmm.12286

**Published:** 2014-04-30

**Authors:** Feng Zhang, Zili Zhang, Li Chen, Desong Kong, Xiaoping Zhang, Chunfeng Lu, Yin Lu, Shizhong Zheng

**Affiliations:** a Department of Pharmacology, College of Pharmacy, Nanjing University of Chinese MedicineNanjing, China; b Jiangsu Key Laboratory for Pharmacology and Safety Evaluation of Chinese Materia Medica, Nanjing University of Chinese MedicineNanjing, China; c National First-Class Key Discipline for Traditional Chinese Medicine of Nanjing University of Chinese MedicineNanjing, China

**Keywords:** liver fibrosis, angiogenesis, hepatic stellate cell, curcumin, peroxisome proliferator-activated receptor-γ, VEGF

## Abstract

Hepatic fibrosis is concomitant with sinusoidal pathological angiogenesis, which has been highlighted as novel therapeutic targets for the treatment of chronic liver disease. Our prior studies have demonstrated that curcumin has potent antifibrotic activity, but the mechanisms remain to be elucidated. The current work demonstrated that curcumin ameliorated fibrotic injury and sinusoidal angiogenesis in rat liver with fibrosis caused by carbon tetrachloride. Curcumin reduced the expression of a number of angiogenic markers in fibrotic liver. Experiments *in vitro* showed that the viability and vascularization of rat liver sinusoidal endothelial cells and rat aortic ring angiogenesis were not impaired by curcumin. These results indicated that hepatic stellate cells (HSCs) that are characterized as liver-specific pericytes could be potential target cells for curcumin. Further investigations showed that curcumin inhibited VEGF expression in HSCs associated with disrupting platelet-derived growth factor-β receptor (PDGF-βR)/ERK and mTOR pathways. HSC motility and vascularization were also suppressed by curcumin associated with blocking PDGF-βR/focal adhesion kinase/RhoA cascade. Gain- or loss-of-function analyses revealed that activation of peroxisome proliferator-activated receptor-γ (PPAR-γ) was required for curcumin to inhibit angiogenic properties of HSCs. We concluded that curcumin attenuated sinusoidal angiogenesis in liver fibrosis possibly by targeting HSCs *via* a PPAR-γ activation-dependent mechanism. PPAR-γ could be a target molecule for reducing pathological angiogenesis during liver fibrosis.

## Introduction

Hepatic fibrosis is caused by excessive extracellular matrix (ECM) deposition concomitant with profound derangements in sinusoidal vascular structure [1]. Current paradigms establish a pivotal role for hepatic stellate cells (HSCs) in liver fibrosis because of their transdifferentiation into myofibroblastic cells that are characterized by enhanced proliferation, motility and capacity to produce ECM [2]. Hepatic stellate cells are also identified as liver-specific pericytes contributing to angiogenesis and vascular remodelling in hepatic fibrogenesis [3]. Existing evidence reveals that HSC secretion of angiogenic cytokines, which stimulate liver sinusoidal endothelial cells (LSECs) *via* paracrine signalling, and recruitment to vascular wall underlie HSC-driven sinusoidal vascular remodelling [4]. These vascular functions of HSCs have been intimately linked to liver fibrosis.

Many signalling pathways mediate the interactions between pericytes and endothelial cells during angiogenesis. In liver, HSCs in close juxtaposition with LSECs can respond to the vascular factors secreted by LSECs. Platelet-derived growth factor (PDGF) produced by LSECs stimulates HSCs to express pro-angiogenic molecules. And as the most motogenic responses in pericyte recruitment to new vessels, PDGF regulates HSC migration and enhances the capability of HSCs to effectively align themselves around vessel wall, thereby promoting pericyte-based sinusoidal vascular remodelling [5]. However, the precise molecular mechanisms remain obscure. Extracellular signal-regulated kinase (ERK) and mammalian target of rapamycin (mTOR) pathways control numerous cellular functions including proliferation, metabolism and survival. Studies have linked the role of ERK or mTOR to VEGF expression during tumour angiogenesis [6,7]. Moreover, focal adhesion kinase (FAK) is known as a key regulator of cell migration in many cell types implicated in various pathophysiological contexts [8]. However, the roles for these pathways in HSC-driven sinusoidal vascularization in fibrosis have not been explored adequately.

Basic and clinical evidence has indicated the tight association between attenuation of angiogenesis and regression of liver fibrosis impulsive for development of potential antifibrotic therapies [4]. We previously reported that curcumin, the polyphenolic pigment in curry from turmeric, protected the liver from carbon tetrachloride (CCl_4_)-caused injury by attenuating oxidative stress and suppressing inflammation in rats [9] and disrupted transforming growth factor-β (TGF-β) signalling leading to inhibition of HSC activation *in vitro* [10]. We also demonstrated that activation of peroxisome proliferator-activated receptor-γ (PPAR-γ) was a prerequisite for curcumin reduction in HSC activation [11]. Our latest investigations showed that activation of PPAR-γ interrupted the FAK/RhoA, ERK and mTOR cascades and inhibited HSC-based vascularization by inhibition of PDGF-β receptor (PDGF-βR) expression [12]. Moreover, curcumin has also been demonstrated to play an anti-angiogenic role in inhibition of tumours [13]; and a recent study showed that curcumin ameliorated sinusoidal capillarization in rat fibrotic liver [14]. However, the underlying molecular mechanism is largely unknown. The current study established a CCl_4_-caused fibrosis model in rats to examine the relevance of sinusoidal angiogenesis attenuation to liver fibrosis reduction by curcumin. And then the mechanisms by which curcumin affected the VEGF expression, cell motility and related vascularization in HSCs were elucidated. The obtained results provided novel insight into the mechanisms of curcumin reduction in hepatic fibrosis.

## Materials and methods

### Reagents and antibodies

Curcumin, Y15, 15d-PGJ2 and PD68235 were obtained from Sigma-Aldrich (St Louis, MO, USA). U0126 was obtained from Cell Signaling Technology (Danvers, MA, USA). Imatinib and fasudil were obtained from Nanjing EnoGene Biotechnology (Nanjing, China). Rapamycin was obtained from Xi'an Helin Biological Engineering (Xi'an, China). All these compounds were dissolved in dimethylsulfoxide (DMSO; Sinopharm Chemical Reagent Co., Ltd., Shanghai, China) for experiments. Recombinant rat PDGF was obtained from Cell Sciences (Canton, MA, USA). Primary antibodies against VEGF, p-PI3K, PI3K, p-AKT and AKT were obtained from Nanjing EnoGene Biotechnology (Nanjing, China). Primary antibodies against α-smooth muscle actin (α-SMA), α(I) procollagen, fibronectin, p-PDGF-βR, PDGF-βR, p-FAK, FAK, GTP-RhoA and total-RhoA were obtained from Epitomics (San Francisco, CA, USA). Primary antibodies against PPAR-γ, p-ERK and ERK were obtained from Cell Signaling Technology. Primary antibodies against HIF-1α, VEGF-R2, p-mTOR, mTOR, p-p70S6K, p70S6K and β-actin were obtained from Bioworld Technology (Nanjing, China).

### Experimental animal procedures

All experimental procedures were approved by the institutional and local committee on the care and use of animals of Nanjing University of Chinese Medicine (Nanjing, China), and all animals received humane care according to the National Institutes of Health (USA) guidelines. Male Sprague-Dawley rats (180–220 g bodyweight) were obtained from Nanjing Medical University (Nanjing, China). A mixture of CCl_4_ (0.1 ml/100 g bodyweight) and olive oil [1:1 (v/v)] was used to induce liver fibrosis in rats. Thirty rats were randomly divided into five groups (six rats/group). Group 1 was the vehicle control in which rats were not administrated CCl_4_ or curcumin but intraperitoneally (i.p.) injected with olive oil. Group 2 was the CCl_4_ group in which rats were i.p. injected with CCl_4_ without curcumin treatment. Groups 3, 4 and 5 were treatment groups in which rats were i.p. injected with CCl_4_ and orally given curcumin at 100, 200 and 400 mg/kg, respectively. Rats in groups 2–5 were i.p. injected with CCl_4_ every other day for 8 weeks. Curcumin was suspended in sterile PBS and given once daily by gavage during weeks 5–8. The control animals in groups 1 and 2 were similarly handled, including i.p. injection with the same volume of olive oil and oral administration of the same volume of PBS. At the end of experiment, rats were weighted and killed after being anaesthetized by i.p. pentobarbital (50 mg/kg). Blood was collected, and livers were isolated for calculation of liver/bodyweight ratio. A small portion of the liver was removed for histopathological and immunohistochemical studies by fixation with 10% formalin and subsequent embedment with paraffin. The remaining liver was cut in pieces and rapidly frozen with liquid nitrogen for extraction of total RNA and hepatic proteins.

### Liver histopathology

Harvested liver tissues were fixed in 10% neutral buffered formalin and embedded in paraffin. Liver slices of 5 μm thick were prepared and stained with haematoxylin and eosin and masson's trichrome stain by using standard methods. For sirius red collagen staining, thin sections were deparaffinized and stained with picro-sirius red for 1 hr at room temperature. After washes, sections on the slides were dehydrated in 100% ethanol and in xylene, and then they were mounted in Permount. Photographs were taken in a blinded fashion at random fields. Representative views of liver sections are shown.

### Hydroxyproline examination

The hydroxyproline levels in liver tissue and blood were determined by using a kit (Nanjing Jiancheng Bioengineering Institute, Nanjing, China) according to the protocol. Briefly, three small pieces of liver tissues randomly excised from the liver of every rat were hydrolyzed in 6 N HCl at 110°C for 24 hrs, and subsequently they were neutralized with NaOH. Isopropanol in citrate acetate-buffered chloramine T was added to aliquots of the hydrolysate, followed by the addition of Ehrlich reagent. The chemical reaction occurred in dark for 25 min. at 60°C. After centrifugation, absorbance of the supernatant of each sample was read at 558 nm by using a 96-well plate spectrometer. Trans-hydroxyproline was used as the standard for quantification. Values were normalized to control.

### Aortic ring assay

Aortas were excised from the thoracic region of healthy male rats and immediately placed in ice-cold PBS. The fat tissue was removed atraumatically, and the aortas were subsequently cut into 0.3 mm rings with a dissecting microscope. The rings were then placed in 100 μl of growth factor reduced matrigel (BD Biosciences, Bedford, MA, USA) in 6-well plates and incubated at 37°C in a humidified 5% CO_2_ incubator for gelation. The rings were incubated in media with various reagents as indicated in specific experiments. The plates were incubated at 37°C in a humidified 5% CO_2_ incubator for 7 days. The rings were fixed in 4% formaldehyde. Photographs of the rings were captured with a phase contrast microscope with a Leica Qwin System (Leica, Wetzlar, Germany). Morphometric analysis of sprouting specifically within the vessel ring lumen was quantified with Image Pro software (Media Cybernetics, Bethesda, MD, USA). Representative micrographs are shown.

### Cell culture

Primary HSCs were isolated from male Sprague-Dawley rats as we previously described in detail [15]. Hepatic stellate cells were cultured in DMEM (Invitrogen, Grand Island, NY, USA) with 10% foetal bovine serum (FBS; Sijiqing Biological Engineering Materials, HangZhou, China), 1% antibiotics and grown in a 5% CO_2_ humidified atmosphere at 37°C. Hepatic stellate cells at passages 2–4 were used in experiments. Primary rat LSECs were purchased from Jiangyin CHI Scientific Inc. (Wuxi, China) and were cultured in endothelial cell culture medium (ScienCell, Carlsbad, CA, USA) with 10% FBS, 1% antibiotics and 10% endothelial cell growth supplement.

### Cell transfection with PPAR-γ siRNA

Peroxisome proliferator-activated receptor-γ siRNA (sc-156077; Santa Cruz Biotechnology, Santa Cruz, CA, USA) of 5 μg was added to 100 μl medium without serum and antibiotics and incubated at room temperature for 5 min. Transfection reagent EnoGeneFec™ 2000 (Nanjing EnoGene Biotechnology) of 25 μl was added to 75 μl medium without serum and antibiotics and incubated at room temperature for 5 min. The above two solutions were mixed well at room temperature for 20 min. and about 200 μl transfection complex was obtained. Then the medium of 800 μl without serum and antibiotics was added to the 200 μl transfection complex and mixed well, and the transfection complex solution of 1000 μl was obtained. Cells were incubated with the transfection complex solution at 37°C for 8 hrs, and then were re-incubated in complete medium at 37°C for an additional 16 hrs. Control siRNA (sc-37007; Santa Cruz Biotechnology) is a non-targeting 20–25 nt siRNA designed as a negative control.

### Cell viability and cytotoxicity assays

Hepatic stellate cells (50,000 cells/well) or LSECs (50,000 cells/well) were seeded in 96-well plates and cultured in DMEM or endothelial cell medium with 10% FBS for 24 hrs. Cells were treated with various reagents as indicated in specific experiments for 24 or 48 hrs. After treatment, 3-(4,5-dimethylthiazol-2-yl)-5-(3-carboxymethoxyphenyl)-2-(4-sulfo-phenyl)-2H-tetrazolium (MTS; Sigma-Aldrich) and phenazine methosulphate (Promega Corporation, Madison, WI, USA) were added and the cells were further incubated for 3 hrs at 37°C. The spectrophotometric absorbance at 490 nm was measured by a SPECTRAmax™ microplate spectrophotometer (Molecular Devices, Sunnyvale, CA, USA). For cytotoxicity assay, lactate dehydrogenase (LDH) activity in culture medium was determined with a LDH release assay kit (Nanjing Jiancheng Bioengineering Institute) according to the protocol.

### Tubulogenesis assay

Low-serum medium (0.5%, v/v) cultured LSECs (100,000 cells/well) or HSCs (50,000 cells/well) were seeded on growth factor reduced matrigel (BD Biosciences) after 30 min. of pre-incubation at 37°C in 48-well plates. Cells were treated with various reagents as indicated in specific experiments or transfected with PPAR-γ siRNA for 24 hrs. Meanwhile, to evaluate the pro-angiogenic effect of treated HSCs on LSECs, HSC conditioned medium was prepared according to described method [16] with minor modification. Briefly, low-serum medium (0.5%, v/v) cultured HSCs (500,000 cells/well) in 6-well plates were treated with reagents for 3 hrs or transfected with PPAR-γ siRNA for 12 hrs. Next, HSCs were grown in fresh serum-free medium for an additional 24 hrs. Conditioned medium were aliquoted and stored at −80°C for further utilization. Liver sinusoidal endothelial cells (100,000 cells/well) were seeded on matrigel in 48-well plates and incubated with mixture medium containing endothelial cell medium and HSC conditioned medium (1:1, v/v). Tubulogenesis was visualized at five random fields per well by using an inverted microscope with a Leica Qwin System (Leica). Tube length was assessed by counting the number of closed intercellular compartments (closed rings or pro-angiogenic structures) by using Image J. Representative micrographs are shown.

### Immunofluorescence staining

After deparaffin, thin sections (5 μm) of the liver tissues were blocked with 1% bovine serum albumin, and then they were incubated with the primary antibodies overnight at 4°C. After three washes with PBS, sections on slides were incubated with fluorescence-conjugated secondary antibodies (Invitrogen, Carlsbad, CA, USA) at room temperature for 2 hrs. Sections incubated with secondary antibodies alone were used as negative controls. For staining with cells, HSCs (50,000 cells/well) were seeded in 6-well plates and cultured in DMEM with 10% FBS for 24 hrs and then in low-serum medium (0.5%, v/v) for an additional 12 hrs. Hepatic stellate cells were then treated with various reagents as indicated in specific experiments for 24 hrs. Cells were incubated with the primary and secondary antibodies in succession similar to the above described protocol. Finally, hoechst 33342 reagent (Beyotime Institute of Biotechnology, Haimen, China) was used to stain the nucleus. Representative micrographs are shown.

### Enzyme-linked immunosorbent assay (ELISA)

The VEGF levels in liver tissues, blood and HSC culture supernatant were determined with an ELISA kit (Nanjing Jiancheng Bioengineering Institute) according to the protocol. For the experiments *in vitro*, HSCs (500,000 cells/well) were seeded in 6-well plates and cultured in DMEM with 10% FBS for 24 hrs and then in low-serum medium (0.5%, v/v) for an additional 12 hrs. Hepatic stellate cells were treated with various reagents as indicated in specific experiments for 24 hrs. Briefly, samples of 100 ml were added to each well of the 96-well plates coated with antibody, followed by incubation for 2 hrs at room temperature. Working detector solution of 100 μl was loaded into each well, and the plates were incubated for an additional 1 hr at room temperature before the addition of substrate solution of 100 μl. The reaction was stopped by adding stop solution of 50 μl. The absorbance was read at 450 nm wavelength. Values were normalized to control.

### Boyden chamber assay

Polycarbonate membrane transwell inserts (8 μm-pore-size; Corning Life Sciences, Corning, NY, USA) were coated with type I collagen (50 μg/ml). Hepatic stellate cells (50,000 cells/well) treated with various reagents as indicated in specific experiments or transfected with PPAR-γ siRNA were suspended in low-serum media (0.5%, v/v) and seeded to the upper wells. The lower chambers were filled with serum-free medium containing vehicle or PDGF (20 ng/ml). After 24 hrs incubation, the polycarbonated filter was removed and migrated cells on the lower surface were counted at random fields. Values were normalized to control.

### Real-time PCR

Total RNA was extracted from frozen liver tissues or treated HSCs by using Trizol reagent according to the protocol provided by the manufacturer (Sigma-Aldrich). Total RNA (1 μg) was treated with DNase I to eliminate genomic DNA contamination followed by synthesis of the first strand by using reverse transcription system (Promega). Reverse transcription was carried out as follows: 42°C for 30 min., 95°C for 5 min. and 4°C for 5 min. (one cycle). Real-time PCR was performed in a 25 μl of reaction solution containing 12.5 μl of 2× iQSYBR Green Supermix (Bio-Rad Laboratories, Hercules, CA, USA), 300 nM primer, and cDNAs. The cycles for PCR were as follows: 95°C for 7 min., 40 cycles of 95°C for 20 sec., 54°C for 30 sec. and 72°C for 30 sec. Melting curves were determined by heat-denaturing PCR products over a 35°C temperature gradient at 0.2°C/s from 60 to 95°C. Glyceraldehyde phosphate dehydrogenase (GAPDH) was used as the invariant control. Fold changes in the mRNA levels of target genes related to the invariant control GAPDH were calculated as suggested [17]. Primer sequences were listed as follows: α-SMA: (forward) 5′-CCGACCGAATGCAGAAGGA-3′, (reverse) 5′-ACAGAGTATTTGCGCTCCGGA-3′; α(I)procollagen: (forward) 5′-CCTCAAGGGCTCCAACGAG-3′, (reverse) 5′-TCAATCACTGTCTTGCCCCA-3′; fibronectin: (forward) 5′-TGTCACCCACCACCTTGA-3′, (reverse) 5′-CTGATTGTTCTTCAGTGCGA-3′; PPAR-γ: (forward) 5′-ATTCTGGCCCACCAACTTCGG-3′, (reverse) 5′-TGGAAGCCTGATGCTTTATCCCCA-3′; HIF-1α: (forward) 5′-TAGACTTGGAAATGCTGGCTCCCT-3′, (reverse) 5′-TGGCAGTGACAGTGATGGTAGGTT-3′; VEGF-R2 (forward) 5′-AGTGGCTAAGGGCATGGAGTTCTT-3′, (reverse) 5′-GGGCCAAGCCAAAGTCACAGATTT-3′; PDGF-βR: (forward) 5′-CTGCCACAGCATGATGAGGATTGAT-3′, (reverse) 5′-GCCAGGATGGCTGAGATCACCAC-3′; VEGF: (forward) 5′-GCACTGGACCCTGGCTTTACT-3′, (reverse) 5′-ATGGGACTTCTGCTCTCCTTCTG-3′; GAPDH: (forward) 5′-GGCCCCTCTGGAAAGCTGTG-3′, (reverse) 5′-CCGCCTGCTTCACCACCTTCT-3′.

### Western blot analyses

Total proteins were prepared from liver tissues or treated HSCs, which were lysed with radio-immunoprecipitation assay buffer to which phenylmethylsulfonyl fluoride and/or phosphatase inhibitor was added immediately after addition of the lysis buffer. The protein levels were determined with a BCA assay kit (Pierce Biotechnology, Rockford, IL, USA). Proteins (50 μg/well) were separated by SDS-polyacrylamide gel, transferred to a PVDF membrane (Millipore, Burlington, MA, USA), blocked with 5% skim milk in Tris-buffered saline containing 0.1% Tween 20. Target proteins were detected by corresponding primary antibodies, and subsequently by horseradish peroxidase-conjugated secondary antibodies. Protein bands were visualized by using chemiluminescence reagent (Millipore). Equivalent loading was confirmed by using an antibody against β-actin. The levels of target protein bands were densitometrically determined by using Image J. The variation in the density of bands was expressed as fold changes compared to the control in the blot after normalized to β-actin, or the total proteins in some experiments.

### Statistical analyses

Results were from at least triplicate experiments. Data were presented as mean ± SD and analysed by using SPSS 16.0 software. Significance of difference was determined by one-way anova with the post-hoc Dunnett's test. Values of *P* < 0.05 were considered to be statistically significant.

## Results

### Curcumin attenuates CCl_4_-caused liver fibrosis in rats

**Fig. 1:**
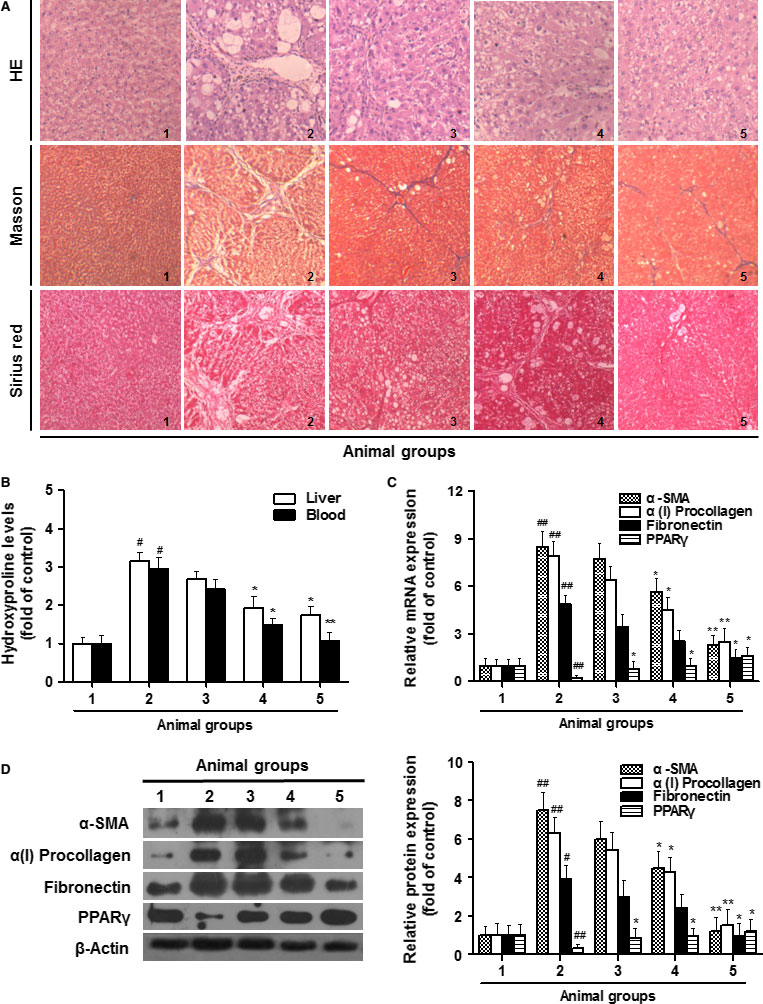
Curcumin attenuates CCl_4_-caused liver fibrosis in rats. Rats were grouped: group 1, vehicle control (no CCl_4_, no treatment); group 2, model group (with CCl_4_, no treatment); group 3, curcumin-treated group (100 mg/kg + CCl_4_); group 4, curcumin-treated group (200 mg/kg + CCl_4_); group 5, curcumin-treated group (400 mg/kg + CCl_4_). (**A**) Liver sections were stained with haematoxylin and eosin, masson reagents and sirius red. (**B**) Measurement of hydroxyproline levels in liver and blood. (**C**) Real-time PCR analyses of α-SMA, α(I)procollagen, fibronectin and peroxisome proliferator-activated receptor-γ in liver tissues. (**D**) Western blot analyses of liver proteins with densitometry. For the statistics of each panel in this figure, ^#^*P* < 0.05 *versus* group 1, ^##^*P* < 0.01 *versus* group 1, *P < 0.05 *versus* group 2, **P < 0.01 *versus* group 2, *n* = 6.

We initially evaluated the curcumin effect on liver fibrotic injury *in vivo*. Curcumin treatment reduced the liver/body weight ratio, which was significantly elevated by CCl_4_ injection (Fig. S1). Curcumin also resulted in remarkable improvement in liver histology and collagen deposition demonstrated by ameliorated state of hepatic steatosis, necrosis and fibrotic septa and by reduced positive-staining size in the liver of CCl_4_-treated rats (Fig. 1A). Measurement of hepatic and blood hydroxyproline further indicated that collagen production was reduced by curcumin in rats with liver fibrosis (Fig. 1B). Furthermore, we examined the transcript and protein abundance of α-SMA, α(1)procollagen, fibronectin, and PPAR-γ, four key markers of liver fibrosis. The results showed that curcumin, at both mRNA and protein levels, downregulated the first three markers but rescued the decreased PPAR-γ expression in fibrotic liver (Fig. 1C and D). Taken together, these data demonstrated that curcumin attenuated CCl_4_-caused liver fibrosis *in vivo*.

### Curcumin alleviates sinusoidal angiogenesis in rat liver with CCl_4_-casused fibrosis possibly by targeting HSCs

**Fig. 2:**
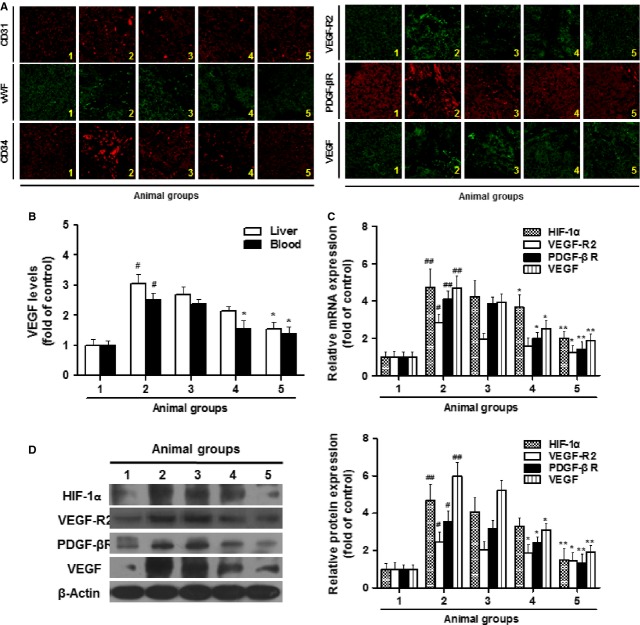
Curcumin alleviates sinusoidal angiogenesis in rats with CCl_4_-caused fibrosis. Rats were grouped: group 1, vehicle control (no CCl_4_, no treatment); group 2, model group (with CCl_4_, no treatment); group 3, curcumin-treated group (100 mg/kg + CCl_4_); group 4, curcumin-treated group (200 mg/kg + CCl_4_); group 5, curcumin-treated group (400 mg/kg + CCl_4_). (**A**) Liver sections were stained with immunofluorescence by using antibodies against CD31, vWF, CD34, VEGF-R2, PDGF-βR, and VEGF. (**B**) ELISA measurement of VEGF levels in liver and blood. (**C**) Real-time PCR analyses of HIF-1α, VEGF-R2, PDGF-βR, and VEGF in liver tissues. (**D**) Western blot analyses of liver proteins with densitometry. For the statistics of each panel in this figure, ^#^*P* < 0.05 *versus* group 1, ^##^*P* < 0.01 *versus* group 1, *P < 0.05 *versus* group 2, **P < 0.01 *versus* group 2, *n* = 6.

We next examined the curcumin effect on fibrosis-associated sinusoidal angiogenesis. As shown in immunofluorescent assay, curcumin decreased the expression of endothelial cell markers (CD31, vWF and CD34) and pro-angiogenic signal molecules (VEGF, VEGF-R2 and PDGF-βR) in fibrotic liver, suggesting that the angiogenic niche involving LSECs and pericytes (HSCs) was alleviated by curcumin (Fig. 2A). VEGF is one of the most potent and central factors that stimulate angiogenesis [18]. In the present study, hepatic and blood levels of VEGF were decreased by curcumin dose-dependently (Fig. 2B). Further experiments demonstrated that the elevated expression of VEGF-R2, PDGF-βR and VEGF in fibrotic liver was abolished by curcumin at both mRNA and protein levels. Noteworthy, hypoxia-inducible factor-1α (HIF-1α) was increased in CCl_4_-caused liver fibrosis but abrogated by curcumin, indicating the alleviation of hepatic hypoxia state (Fig. 2C and D).

**Fig. 3:**
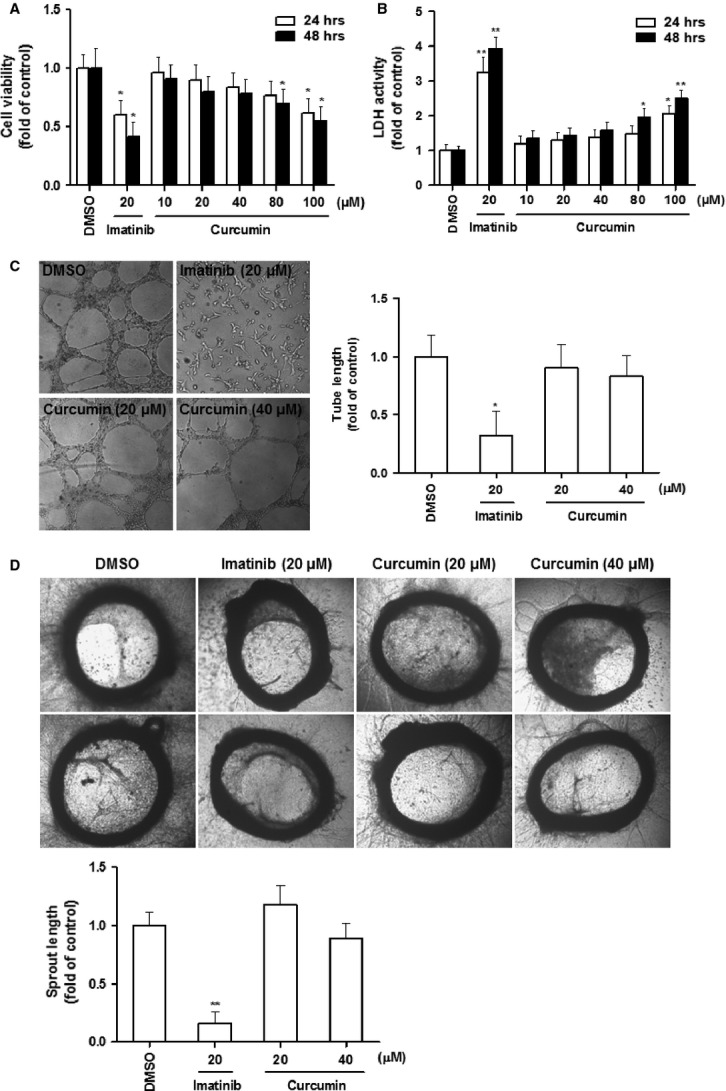
Curcumin does not affect endothelial cells and aortic ring vascularization *in vitro*. (**A** and **B**) LSECs were treated with DMSO (0.02%, w/v), imatinib and curcumin for 24 or 48 hrs. Cell viability was evaluated by MTS assay (**A**). Lactate dehydrogenase activity in supernatant was assessed (**B**). (**C**) LSECs were incubated with DMSO (0.02%, w/v), imatinib and curcumin on matrigel for 24 hrs. Tubulogenesis was visualized and quantified. (**D**) Explanted aortic rings were placed on matrigel and subjected to DMSO (0.02%, w/v), imatinib and curcumin for 7 days (*n* = 6). The histogram shows quantification of aortic sprouts specifically from the endothelial cell lumen. Values are represented as arbitrary units. For the statistics of each panel in this figure, *P < 0.05 *versus* DMSO, **P < 0.01 *versus* DMSO.

Although LSECs paly an obligatory role in angiogenesis including the physiological angiogenesis beneficial for liver regeneration, activated HSCs are the pivotal effecter cells in the pathogenesis of liver fibrosis and associated sinusoidal vascular remoulding [3]. Interestingly, curcumin, within a wide range of doses, did not apparently affect LSEC viability and had no toxicity *in vitro*; however, the angiogenesis inhibitor imatinib at 20 μM reduced LSEC viability with significant cytotoxicity (Fig. 3A and B). In tubulogenesis assay, imatinib completely prevented LSEC-mediated tube formation, but curcumin showed no apparent inhibitory effect (Fig. 3C). Aortic ring assay provided *ex vivo* evidence that curcumin could not affect physiological vascularization (Fig. 3D). Collectively, these data indicated that LSECs might not be the direct target cells for curcumin. By virtue of our prior data on curcumin inhibition of HSC activation [10,19], it could be postulated that pericytes (HSCs) were potential cellular target for curcumin to attenuate pathological angiogenesis in fibrotic liver.

### Curcumin interrupts PDGF-βR/ERK and mTOR pathways linking to reduction in VEGF expression in HSCs

**Fig. 4:**
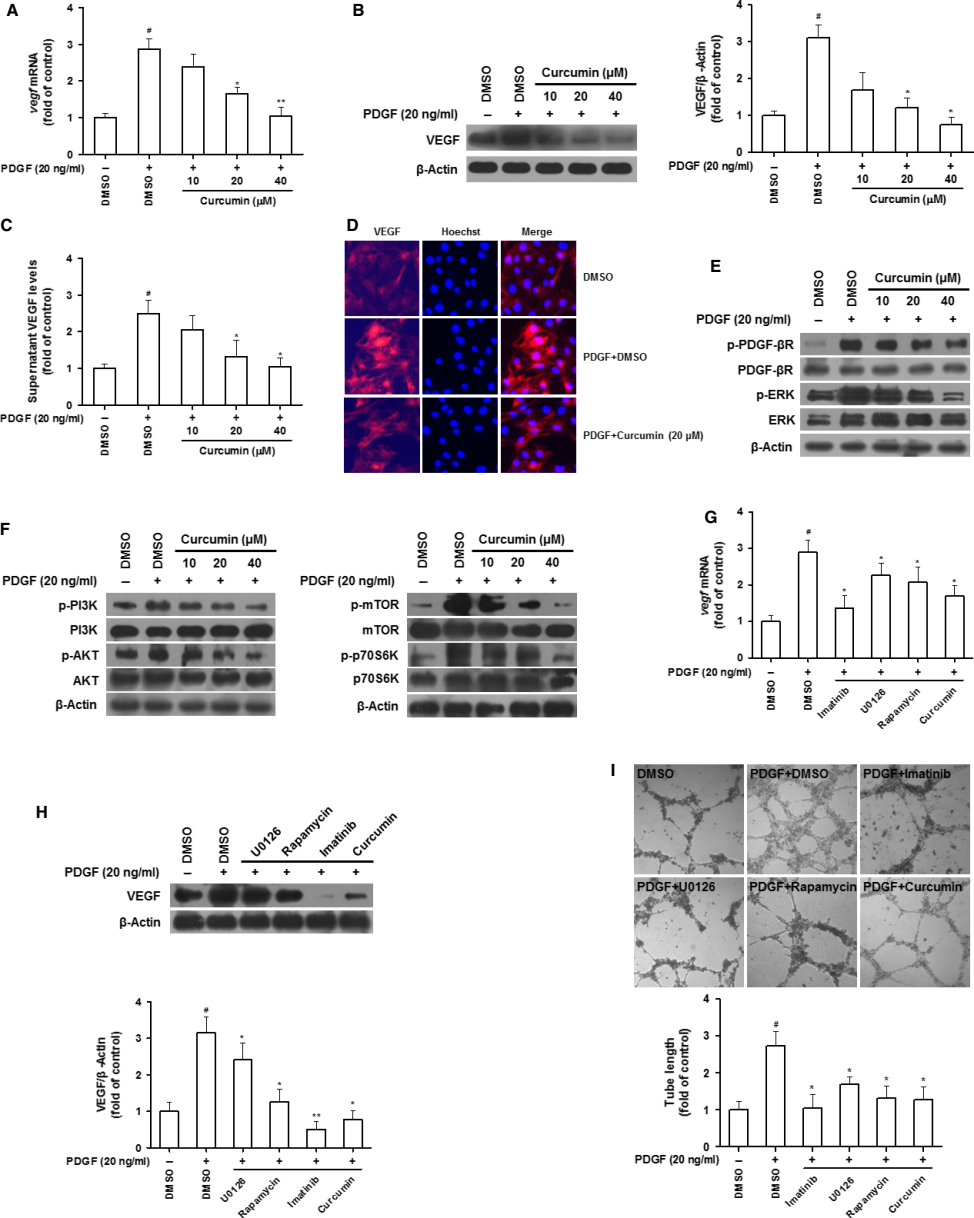
Curcumin interrupts PDGF-βR/ERK and PI3K/AKT/mTOR pathways linking to reduced VEGF expression in HSCs. (**A**–**D**) HSCs were treated with DMSO (0.02%, w/v), PDGF (20 ng/ml) and curcumin for 24 hrs. Real-time PCR analyses of VEGF mRNA (**A**). Western blot analyses of VEGF protein expression with densitometry (**B**). ELISA measurement of VEGF level in supernatant (**C**). Immunofluorescence by using antibody against VEGF (**D**). (**E** and **F**) HSCs were treated with DMSO (0.02%, w/v) and curcumin for 24 hrs prior to PDGF stimulation for an additional 3 hrs. Western blot analyses of PDGF-βR/ERK signals (**E**) and PI3K/AKT/mTOR signals (**F**). (**G** and **H**) HSCs were treated with DMSO (0.02%, w/v), imatinib (10 μM), U0126 (10 μM), rapamycin (10 nM) and curcumin (20 μM) for 24 hrs prior to PDGF stimulation for an additional 3 hrs. Real-time PCR analyses of VEGF mRNA (**G**). Western blot analyses of VEGF protein expression with densitometry (**H**). (**I**) LSECs were incubated with conditioned media from HSCs treated with DMSO (0.02%, w/v), PDGF (20 ng/ml), imatinib (10 μM), U0126 (10 μM), rapamycin (10 nM) and curcumin (20 μM) for 24 hrs. Tubulogenesis was visualized and quantified. For the statistics of each panel in this figure, ^#^*P* < 0.05 *versus* DMSO, ^##^*P* < 0.01 *versus* DMSO, *P < 0.05 *versus* DMSO + PDGF, **P < 0.01 *versus* DMSO + PDGF.

We then investigated the curcumin impact on angiogenic properties of HSCs and the underlying mechanisms. Since PDGF is excessively produced in fibrotic liver functioning as the most potent mitogenic molecule responsible for HSC activation [20], this stimulus was used in our subsequent experiments mimicking the *in vivo* status of HSCs. Additionally, cell viability assay provided a rationale for curcumin doses used in subsequent *in vitro* experiments (Fig. S2A). VEGF is a predominant angiogenic stimulus during liver fibrosis [21]. Our data demonstrated that the increased VEGF expression in response to PDGF was abrogated by curcumin dose-dependently at both mRNA and protein levels (Fig. 4A and B). This result was confirmed by measuring VEGF levels in HSC supernatant (Fig. 4C) and immunofluorescence staining with VEGF antibody (Fig. 4D). We postulated that PDGF-βR/ERK and PI3K/AKT/mTOR pathways could be involved in curcumin effect. Our results showed that the PDGF-enhanced phosphorylation of PDGF-βR, ERK, PI3K, AKT and mTOR, as well as p70S6K, a primary downstream molecule of mTOR, was diminished by curcumin dose-dependently, suggesting disruption of the two pathways (Fig. 4E and F). Subsequently, PDGF-βR inhibitor imatinib, ERK inhibitor U0126 and mTOR inhibitor rapamycin were used to establish the link between curcumin disruption of the two pathways and reduction in VEGF expression. MTS assays indicated appropriate doses of the three inhibitors, respectively, at which cell viability was not affected but intracellular signalling events may be modulated (Fig. S2B–D). Interestingly, all the three inhibitors at the selected doses significantly abolished PDGF-increased VEGF mRNA and protein expression mimicking the curcumin effect (Fig. 4G and H). Furthermore, we postulated that VEGF expression contributed to the ability of HSCs to stimulate angiogenesis. We thus evaluated the effect of HSC conditional media on LSEC-mediated tube formation. Liver sinusoidal endothelial cells incubated with the conditional media of HSCs treated with the three inhibitors and curcumin showed remarkably attenuated vascularization (Fig. 4I). Altogether, these data indicated that curcumin reduced VEGF expression associated with disruption of PDGF-βR/ERK and mTOR pathways in activated HSCs.

### Curcumin interrupts PDGF-βR/FAK/RhoA pathway linking to inhibition of HSC invasion and vascularization

**Fig. 5:**
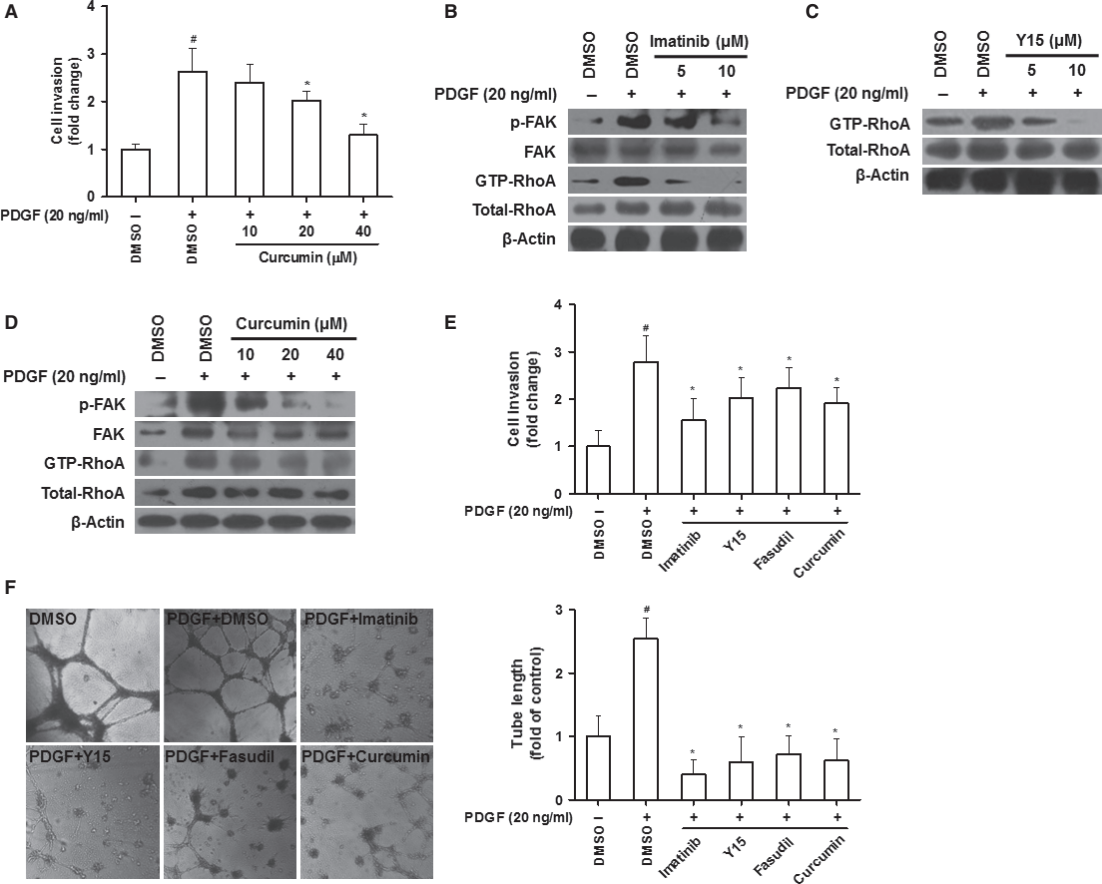
Curcumin interrupts PDGF-βR/FAK/RhoA pathway linking to inhibited HSC invasion and vascularization. (**A**) HSC invasion for 24 hrs was evaluated by Boyden chamber assay with DMSO (0.02%, w/v) and curcumin in the upper well and PDGF in the lower well. (**B**–**D**) HSCs were treated with DMSO (0.02%, w/v), imatinib (**B**), Y15 (**C**), or curcumin (**D**) for 24 hrs prior to PDGF stimulation for an additional 3 hrs. Western blot analyses of FAK/RhoA signals. (**E**) HSC invasion for 24 hrs was evaluated by Boyden chamber assay with DMSO (0.02%, w/v), imatinib (10 μM), Y15 (10 μM), fasudil (10 μM) and curcumin (20 μM) in the upper well and PDGF in the lower well. (**F**) HSCs were incubated with DMSO (0.02%, w/v), PDGF (20 ng/ml), imatinib (10 μM), Y15 (10 μM), fasudil (10 μM) and curcumin (20 μM) on matrigel for 24 hrs. Tubulogenesis was visualized and quantified. For the statistics of each panel in this figure, ^#^*P* < 0.05 *versus* DMSO, *P < 0.05 *versus* DMSO + PDGF.

Enhanced HSC motility contributes to HSC-driven angiogenesis during liver fibrosis [4]. Curcumin reduced the PDGF-enhanced HSC invasion dose-dependently (Fig. 5A). Evidence suggests that cell motility may be regulated by FAK and RhoA [7,22]. In the present study, imatinib reduced FAK phosphorylation and GTP-bound RhoA (Fig. 5B), and FAK inhibitor Y15 diminished GTP-bound RhoA (Fig. 5C), indicating a PDGF-βR/FAK/RhoA cascade in activated HSCs. Curcumin dose-dependently decreased FAK phosphorylation and GTP-bound RhoA (Fig. 5D). The link of disruption of PDGF-βR/FAK/RhoA signalling to curcumin reduction in HSC motility was further established by the observation that pharmacological inhibition of PDGF-βR, FAK and RhoA by their specific inhibitors imatinib, Y15 and fasudil, respectively, abrogated the increased HSC invasion in response to PDGF, and that curcumin produced similar reducing effect (Fig. 5E). The used doses for Y15 and fasudil were also determined by additional MTS assays (Fig. S3A and B). Given that HSCs as liver pericytes have angiogenic capacity [3], tubulogenesis assay was performed to examine HSC tube formation *in vitro*. PDGF-stimulated tubulogenesis in HSCs was abolished by imatinib, Y15, fasudil and curcumin, indicating that curcumin inhibition of HSC vascularization could be associated with blockade of PDGF-βR/FAK/RhoA signalling (Fig. 5F). Taken together, our findings suggested that curcumin interrupted PDGF-βR/FAK/RhoA cascade relevant to inhibition of HSC invasion and vascularization.

### Activation of PPAR-γ is required for curcumin inhibition of angiogenic properties of HSCs

**Fig. 6:**
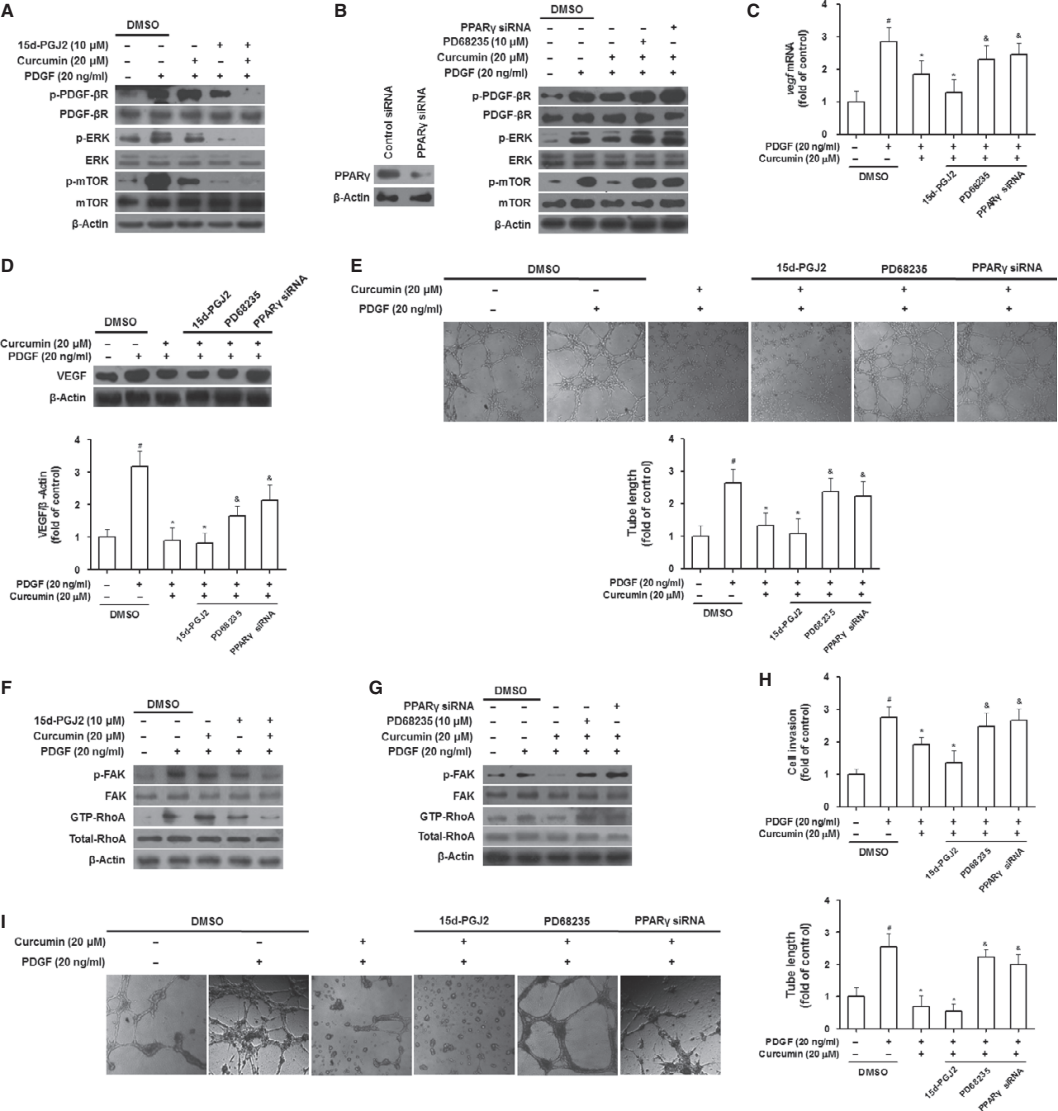
Peroxisome proliferator-activated receptor-γ activation is required for curcumin inhibition of angiogenic properties of HSCs. (**A** and **B**) HSCs were treated with DMSO (0.02%, w/v), curcumin, 15d-PGJ2 (**A**), or PD68235 or transfected with siRNA (**B**) for 24 hrs prior to PDGF stimulation for an additional 3 hrs. Western blot analyses of PDGF-βR/ERK and mTOR signals. (**C** and **D**) HSCs were treated with DMSO (0.02%, w/v), PDGF, curcumin, 15d-PGJ2 (10 μM), PD68235 (10 μM) or transfected with siRNA for 24 hrs. Real-time PCR analyses of VEGF mRNA (**C**). Western blot analyses of VEGF protein expression with densitometry (**D**). (**E**) LSECs were incubated with conditioned media from HSCs treated with DMSO (0.02%, w/v), PDGF, curcumin, 15d-PGJ2 (10 μM), PD68235 (10 μM), or transfected with siRNA for 24 hrs. Tubulogenesis was visualized and quantified. (**F** and **G**) HSCs were treated with DMSO (0.02%, w/v), curcumin, 15d-PGJ2 (**F**), or PD68235 or transfected with siRNA (**G**) for 24 hrs prior to PDGF stimulation for an additional 3 hrs. Western blot analyses of FAK/RhoA signals. (**H**) HSC invasion for 24 hrs was evaluated by Boyden chamber assay with DMSO (0.02%, w/v), curcumin, 15d-PGJ2 (10 μM), PD68235 (10 μM), or transfection with siRNA in the upper well and PDGF in the lower well. (**I**) HSCs were treated with DMSO (0.02%, w/v), PDGF, curcumin, 15d-PGJ2 (10 μM), PD68235 (10 μM), or transfected with siRNA for 24 hrs. Tubulogenesis was visualized and quantified. For the statistics of each panel in this figure, ^#^*P* < 0.05 *versus* DMSO, *P < 0.05 *versus* DMSO + PDGF, ^&^*P* < 0.05 *versus* curcumin + PDGF.

Peroxisome proliferator-activated receptor-γ has been established as a target molecule for curcumin suppression of HSC activation [23]. Herein, we postulated that activation of PPAR-γ could also be a prerequisite for curcumin to inhibit angiogenic properties of HSCs. Gain- or loss-of-function analyses were employed to test the hypothesis. Peroxisome proliferator-activated receptor-γ agonist 15d-PGJ2 [19], similar to curcumin, reduced phosphorylation of PDGF-βR, ERK and mTOR (Fig. 6A); however, PPAR-γ antagonist PD68235 [24] and siRNA-mediated PPAR-γ knockdown rescued curcumin inhibition of these signals (Fig. 6B). Curcumin reduction in VEGF mRNA and protein expression was strengthened by 15d-PGJ2, but rescued by PD68235 and PPAR-γ siRNA (Fig. 6C and D). The LSEC tubulogenesis assay with HSC conditional media showed that 15d-PGJ2 reinforced curcumin suppression of HSC ability to stimulate angiogenesis, but PD68235 and PPAR-γ siRNA abrogated curcumin effect (Fig. 6E). These data indicated that activation of PPAR-γ was required for curcumin to interrupt PDGF-βR/ERK and mTOR pathways and thereby to inhibit VEGF expression in activated HSCs. Furthermore, we investigated the role of PPAR-γ in curcumin inhibition of FAK/RhoA pathway-mediated HSC motility and vascularization. Ligand activation of PPAR-γ by 15d-PGJ2 blocked FAK/RhoA signalling and inhibited HSC invasion and tuber formation, mimicking curcumin effects; however, PD68235 or siRNA-mediated genetic knockdown of PPAR-γ rescued curcumin reduction in FAK/RhoA-mediated HSC motility and vascularization in activated HSCs (Fig. 6F–I). These findings collectively indicated that curcumin inhibited angiogenic properties of HSCs through a PPAR-γ activation-dependent mechanism.

## Discussion

Recent studies have highlighted intrahepatic angiogenesis and sinusoidal remodelling as novel targets for the treatment of liver fibrosis [3,4]. In the present study, we demonstrated that curcumin protected the rat liver from CCl_4_-caused injury and fibrogenesis, which was in agreement with the prior data reported by our group [9] and others [25–27]. Moreover, we clearly showed that curcumin inhibited intrahepatic vascularization in rat fibrotic liver. Examination of several key angiogenic markers demonstrated downregulation of endothelial cell markers and angiogenic signal molecules, suggesting that curcumin reduced the microvessel density and inhibited the pro-angiogenic pathways that aggravate the angiogenic niche in fibrotic liver. Noteworthy, curcumin reduced HIF-1α expression indicative of correction of tissue hypoxia. The hypoxia in fibrotic liver has been attributed to the structural changes in the sinusoids including basement membrane deposition and loss of LSEC fenestrae, which in turn leads to impaired oxygen diffusion from the sinusoids to the parenchyma [28]. Our *in vivo* data were in good agreement with a recent study where curcumin similarly suppressed multiple pro-angiogenic factors including vWF, CD31, VEGFR-1, VEGFR-2, placental growth factor and cyclooxygenase-2 in rat fibrotic liver [14].

Both endothelial cells and pericytes play obligatory roles in angiogenesis. Physiological angiogenesis occurring in liver regeneration is beneficial for recovery from liver injury [29]. We herein revealed that curcumin did not apparently affect LSEC viability and vascularization as well as aortic vessel growth in a wide range of doses, suggesting that curcumin might not affect physiological angiogenesis during hepatic fibrosis. This could be supported by the recognition that curcumin is a hepatoprotective agent without toxic effects on hepatocytes [30]. These results were also in agreement with previous reports that curcumin could protect endothelial cells and correct endothelial dysfunction under various pathophysiological circumstances [31–35]. Together with our data and others, we extrapolated that curcumin could selectively ameliorate HSC-driven sinusoidal angiogenesis, which is pathological because HSCs are characterized as the pivotal effector cells in the pathogenesis of hepatic fibrosis [2].

We next aimed at elucidating the mechanisms by which curcumin modulated angiogenic properties of HSCs. During neovascularization, VEGF plays a predominant role in the initial stages of new vessel formation, endothelial cell proliferation and the subsequent tubule formation [36]. During liver fibrosis, HSC production of VEGF contributes greatly to HSC-driven sinusoidal vascularization [3]. Our current data showed that curcumin inhibited VEGF expression and disrupted PDGF-βR/ERK and PI3K/AKT/mTOR cascades in activated HSCs. Use of specific inhibitors of key signalling molecules provided evidence that curcumin reduction in VEGF expression and inhibition of HSC-mediated LSEC tube formation were associated with blockade of PDGF-βR/ERK and mTOR pathways. These data could be supported by recent evidence that VEGF production in HSCs was associated with overexpression of cyclooxygenase-2 *via* ERK activation [37] and that activation of PI3K/AKT/mTOR signalling increased VEGF secretion in tumour cells [7]. Furthermore, it is known that mTOR forms two functionally distinct complexes, namely mTOR complex 1 (mTORC1), which is sensitive to rapamycin, and mTORC2 in mammalian cells [38]. Since rapamycin significantly reduced VEGF expression observed in our experiments, it therefore could be presumed that mTORC1 was involved in the mechanisms regulating VEGF expression and curcumin intervention in HSCs. The present study also suggested that disruption of PDGF-βR/FAK/RhoA pathway was linked to curcumin inhibition of HSC motility and angiogenic capacity. Curcumin blockade of FAK or RhoA signalling for inhibition of migration and invasion was also demonstrated in many other cell types [39,40]. Noteworthy, our recent work has established that PDGF-βR/ERK and mTOR pathways governed VEGF expression in HSCs and that PDGF-βR/FAK/RhoA signalling is required for HSC recruitment and vascularization [12], which could strongly support the current speculation that interruption of these angiogenic pathways might play a causal role in curcumin inhibition of HSC angiogenic properties. Although more analyses are needed to firmly confirm the molecular pathways underlying curcumin effects, the present studies highlighted the potential to target these signalling systems for intervention of pathological angiogenesis during chronic liver injury.

**Fig. 7:**
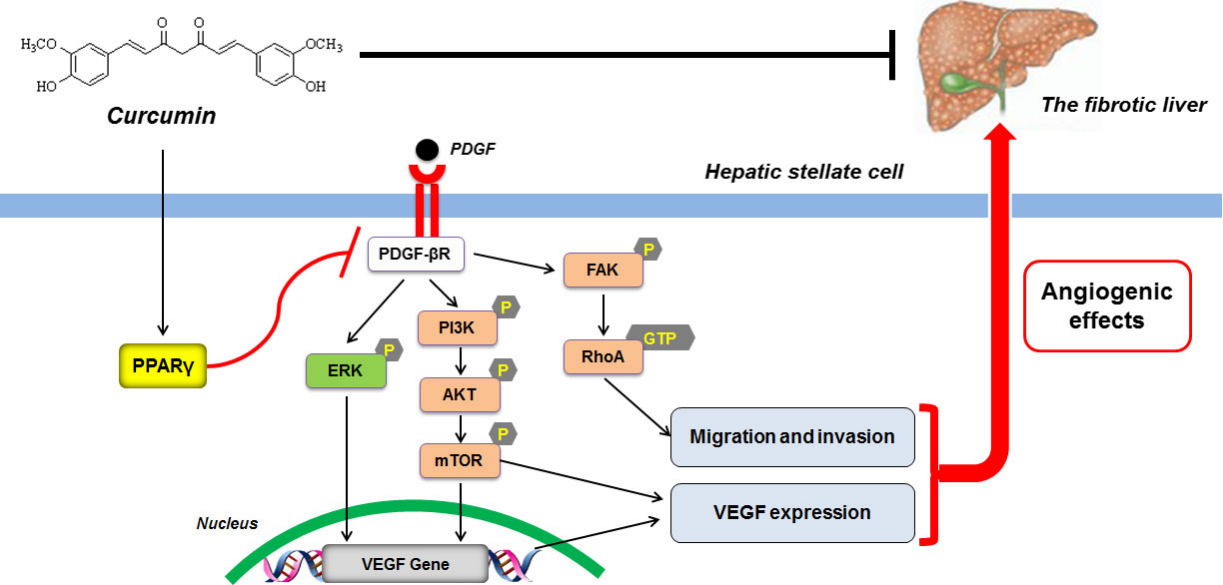
Schema of the underlying mechanism of curcumin inhibition of angiogenic properties of HSCs. Curcumin activates peroxisome proliferator-activated receptor-γ possibly leading to transrepression of PDGF-βR, which disrupts ERK and PI3K/AKT/mTOR pathways and thereby inhibits VEGF mRNA and protein expression in activated HSCs. Inhibition of PDGF-βR also blocks FAK/RhoA cascade resulting in reduced HSC motility. These actions in concert attenuate the angiogenic effects of HSCs. The identified mechanism possibly accounts for curcumin attenuation of HSC-based pathological angiogenesis in liver fibrosis.

Studies over the past decade have implicated the pivotal role for PPAR-γ in the pathogenesis of liver fibrosis. Activation of PPAR-γ can inhibit HSC collagen production and modulate HSC adipogenic phenotype at transcriptional and epigenetic levels [41]. Peroxisome proliferator-activated receptor-γ also cross-regulates a number of signalling pathways mediated by growth factors and adipokines, which are critically associated with liver fibrogenesis [23]. These discoveries illuminate a framework for developing new PPAR-γ modulators with antifibrotic activity. Our prior investigations have identified curcumin as a PPAR-γ activator inhibiting HSC activation [10,11,19]. The present data provided further evidence that curcumin attenuation of sinusoidal pathological angiogenesis could also be dependent on activation of PPAR-γ. We used complementary approaches including pharmacological modulation and siRNA-mediated gene knockdown to convincingly demonstrate the critical role for PPAR-γ in curcumin disruption of PDGF-βR/ERK, mTOR, and FAK/RhoA cascades and suppression of their downstream angiogenic effects in activated HSCs. This was in accordance with the recognition that PDGF-βR acts as an upstream signalling molecule leading to initiation of a number of signal pathways including ERK and PI3K/AKT/mTOR cascades in many cell types [42]. Furthermore, the current data could confirm PPAR-γ transrepression of PDGF-βR in HSCs, a mechanism proposed in our recent study showing that PDGF-βR was a target gene negatively regulated by PPAR-γ and that disruption of PDGF-βR-mediated signal pathways contributed to PPAR-γ inhibition of HSC angiogenic properties [12]. These molecular events could underlie curcumin interruption of angiogenic signal transduction in HSCs. However, it remains unclear whether there are certain transcription factors involved in curcumin effects, because activated PPAR-γ could interfere with the activity and function of some transcription factors such as NF-кB and AP-1, leading to disruption of their binding to target gene promoters and thereby to transcription repression [43,44]. A simplified mode was proposed to describe the mechanisms underlying curcumin blockade of angiogenic cascades and inhibition of angiogenic properties in HSCs (Fig. 7). These molecular discoveries strongly indicated that PPAR-γ could be a target molecule for developing antifibrotic agents used to treat HSC-driven pathological angiogenesis in hepatic fibrosis.

In summary, the present study demonstrated that curcumin ameliorated fibrosis-associated sinusoidal pathological angiogenesis *in vivo* possibly through targeting HSCs. Mechanistic investigations revealed that activation of PPAR-γ was required for curcumin to block PDGF-βR-mediated ERK, mTOR and FAK/RhoA cascades linking to inhibition of angiogenic properties in HSCs. We anticipate that these novel discoveries could be impulsive for developing curcumin as an antifibrotic agent.

## Supporting information

**Figure S1** Curcumin reduces liver/body weight ratio in rat fibrotic liver caused by CCl_4_. Rats were grouped: group 1, vehicle control (no CCl_4_, no treatment); group 2, model group (with CCl_4_, no treatment); group 3, curcumin-treated group (100 mg/kg + CCl_4_); group 4, curcumin-treated group (200 mg/kg + CCl_4_); group 5, curcumin-treated group (400 mg/kg + CCl_4_). ^#^*P* < 0.05 *versus* group 1, **P* < 0.05 *versus* group 2, *n* = 6.

**Figure S2** Effects of curcumin (**A**), imatinib (**B**), U0126 (C) and rapamycin (**D**) on cell viability in HSCs. HSCs were treated with DMSO (0.02%, w/v) and compounds at indicated concentrations for 24 hrs. Cell viability was evaluated by MTS assay. For the statistics of each panel in this figure, **P* < 0.05 *versus* control.

**Figure S3** Effects of Y15 (**A**) and fasudil (**B**) on cell viability in HSCs. HSCs were treated with DMSO (0.02%, w/v) and compounds at indicated concentrations for 24 hrs. Cell viability was evaluated by MTS assay. For the statistics of each panel in this figure, **P* < 0.05 *versus* control.
