# Supplementary figures and images for: Curcumin attenuates angiogenesis in liver fibrosis and inhibits angiogenic properties of hepatic stellate cells
Source: J Cell Mol Med. 2014 Apr 30;18(7):1392–406. doi: 10.1111/jcmm.12286 (PMC4124023; doi:10.1111/jcmm.12286)

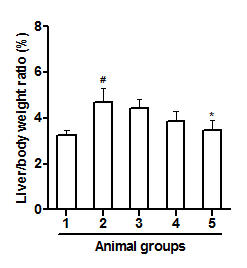

Supplement: Supplementary file 1 — Figure S1 Curcumin reduces liver/body weight ratio in rat fibrotic liver caused by CCl4. Rats were grouped: group 1, vehicle control (no CCl4, no treatment); group 2, model group (with CCl4, no treatment); group 3, curcumin-treated group (100 mg/kg + CCl4); group 4, curcumin-treated group (200 mg/kg + CCl4); group 5, curcumin-treated group (400 mg/kg + CCl4). #P < 0.05 versus group 1, *P < 0.05 versus group 2, n = 6. [file jcmm0018-1392-SD1.tif]

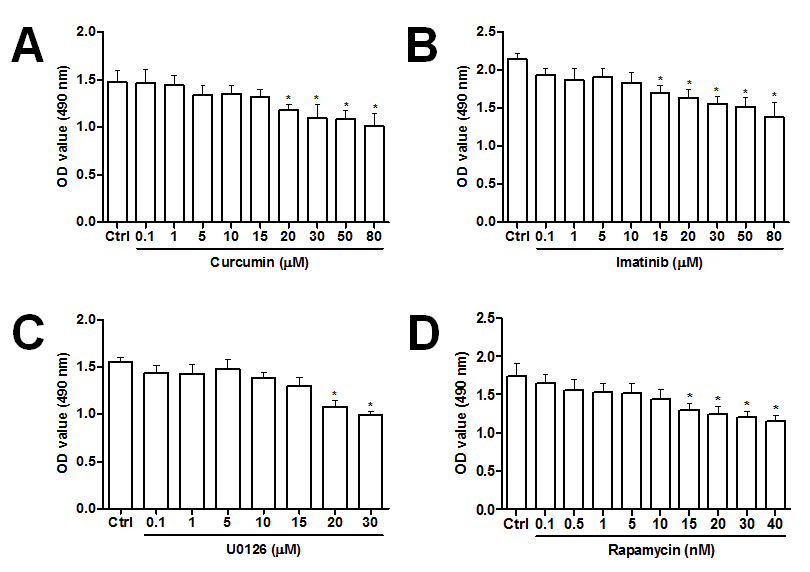

Supplement: Supplementary file 2 — Figure S2 Effects of curcumin (A), imatinib (B), U0126 (C) and rapamycin (D) on cell viability in HSCs. HSCs were treated with DMSO (0.02%, w/v) and compounds at indicated concentrations for 24 hrs. Cell viability was evaluated by MTS assay. For the statistics of each panel in this figure, *P < 0.05 versus control. [file jcmm0018-1392-SD2.tif]

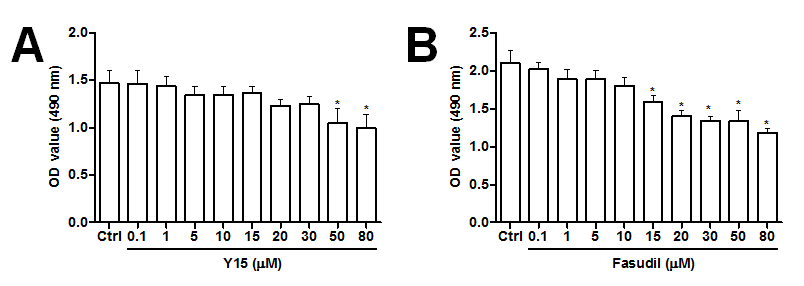

Supplement: Supplementary file 3 — Figure S3 Effects of Y15 (A) and fasudil (B) on cell viability in HSCs. HSCs were treated with DMSO (0.02%, w/v) and compounds at indicated concentrations for 24 hrs. Cell viability was evaluated by MTS assay. For the statistics of each panel in this figure, *P < 0.05 versus control. [file jcmm0018-1392-SD3.tif]
